# Performance of a multiplexed amplicon-based next-generation sequencing assay for HLA typing

**DOI:** 10.1371/journal.pone.0232050

**Published:** 2020-04-23

**Authors:** Chang Liu, Brian F. Duffy, Eric T. Weimer, Maureen C. Montgomery, Jo-Ellen Jennemann, Rachel Hill, Donna Phelan, Lindsay Lay, Bijal A. Parikh

**Affiliations:** 1 Division of Laboratory and Genomic Medicine, Department of Pathology and Immunology, School of Medicine, Washington University in St. Louis, St. Louis, Missouri, United States of America; 2 HLA Laboratory, Barnes-Jewish Hospital, St. Louis, Missouri, United States of America; 3 Department of Pathology & Laboratory Medicine, University of North Carolina at Chapel Hill School of Medicine, Chapel Hill, North Carolina, United States of America; 4 Molecular Immunology Laboratory, McLendon Clinical Laboratories, UNC Hospitals, Chapel Hill, North Carolina, United States of America; Defense Threat Reduction Agency, UNITED STATES

## Abstract

**Background:**

Next-generation sequencing (NGS) has enabled efficient high-resolution typing of human leukocyte antigen (HLA) genes with minimal ambiguity. Most commercially available assays amplify individual or subgroup of HLA genes by long-range PCR followed by library preparation and sequencing. The AllType assay simplifies the workflow by amplifying 11 transplant-relevant HLA genes in one PCR reaction. Here, we report the performance of this unique workflow evaluated using 218 genetically diverse samples.

**Methods:**

Five whole genes (HLA-A/B/C/DQA1/DPA1) and six near-whole genes (HLA-DRB1/DRB345/DQB1/DPB1; excluding exon 1 and part of intron 1) were amplified in a multiplexed, long-range PCR. Manual library preparation was performed per manufacturer’s protocol, followed by template preparation and chip loading on the Ion Chef, and sequencing on the Ion S5 sequencer. Pre-specified rules for quality control and repeat testing were followed; technologists were blinded to the reference results. The concordance between AllType and reference results was determined at 2-field resolution. We also describe the ranges of input DNA and library concentrations, read number per sample and per locus, and key health metrics in relation to typing results.

**Results:**

The concordance rates were 98.6%, 99.8% and 99.9% at the sample (n = 218), genotype (n = 1688), and allele (n = 3376) levels, respectively. Three genotypes were discordant, all of which shared the same G group typing results with the reference. Most ambiguous genotypes (116 out of 144, 80.6%) were due to the lack of exon 1 and intron 1 coverage for HLA-DRB1/DRB345/DQB1/DPB1 genes. A broad range of input DNA concentrations and library concentrations were tolerated. Per sample read numbers were adequate for accurate genotyping. Per locus read numbers showed some inter-lot variations, and a trend toward improved inter-locus balance was observed with later lots of reagents.

**Conclusion:**

The AllType assay on the Ion Chef/Ion S5 platform offers a robust and efficient workflow for clinical HLA typing at the 2-field resolution. The multiplex PCR strategy simplifies the laboratory procedure without compromising the typing accuracy.

## Introduction

Next-generation sequencing (NGS) has been rapidly adopted for HLA typing in clinical laboratories to evaluate donor-recipient histocompatibility. The key advantage of NGS over Sanger sequencing for high-resolution HLA typing is that NGS can phase the large number of sequence variants within HLA genes into haplotypes with minimal cis-trans ambiguity [1–3]. In addition, most current NGS HLA typing assays cover full or near-whole target HLA genes, allowing the detection of clinically relevant variants outside of key exons (exons 2 and 3 of class I genes and exon 2 of class II genes). The high throughput of NGS enables multiplexed sequencing of DNA enriched from multiple genes and many samples in one run, which maximizes the efficiency and lowers the per sample cost.

While sequencing of the whole genome [4], whole exome [5, 6], transcriptome [7, 8], or captured HLA genes can lead to successful HLA typing, most commercially available NGS assays amplify target HLA genes by polymerase chain reaction (PCR) followed by library preparation and sequencing [9–12]. One common approach is the “long-amplicon, short-read” approach. The whole, or near-whole HLA genes are enriched by PCR, and the amplicons are fragmented through enzyme digestion and then ligated to barcodes and sequencing adapters. Short sequencing reads from Illumina and Ion-torrent sequencers are demultiplexed and mapped to reference sequences to generate HLA typing of 2-field, and up to 4-field, resolution. This approach has been shown to achieve highly accurate typing results at a shortened turnaround time [9–12].

For clinical HLA laboratories, workflow efficiency is an important consideration when selecting an NGS-based HLA typing method. We evaluated the performance of the AllType assay (One Lambda, West Hills, CA), which enriches 11 HLA genes (HLA- A/B/C/DRB1/DRB345/DQB1/DQA1/DPB1/DPA1) in one multiplexed PCR reaction followed by library preparation and sequencing on the Ion Chef/Ion S5 platform. All other amplicon-based commercial assays reported in the literature, to our knowledge, amplify individual or subgroups of HLA genes by multiple PCR reactions. It is critical to assess the impact of factors, such as template DNA and library concentrations, inter-locus balance, and allele balance, on the success of this assay. Here, we present the results of our evaluation of the AllType workflow using 218 genetically diverse samples.

## Materials and methods

### Samples and reference typing results

We sequenced 218 unique samples in this study, including 71 de-identified patient samples (67 EDTA- or citrate-anticoagulated peripheral blood, 2 buccal specimens, and 2 bone marrow biopsy specimens), 96 external proficiency test (PT) specimens from three different vendors, and 51 cell line-derived DNA samples from the Sequence Polymorphism (SP) panel of the 13^th^ International Histocompatibility Working Group (IHWG). The study falls outside the purview of the Human Research Protection Office in our institution, because de-identified specimens were tested, no links will enable the re-identification of subjects, and providers of the specimens were not involved in the design or conduct of the research. High-resolution reference typing results (2-field) were available for HLA-A/B/C/DRB1/DQB1 in all samples, For HLA-DPA1/DPB1/DQA1/DRB345, reference typing results were available in 133, 154, 163, and 153 samples, respectively. Reference typing results for local patient samples were generated by Sanger sequencing supplemented by sequence-specific oligonucleotide (SSO) and sequence-specific primer (SSP) methods. Reference results of PT specimens were consensus results among participating laboratories. Reference results of the SP panel were generated by the 13^th^ IHWG and 17^th^ International HLA and Immunogenetics Workshop. A total of 341 unique HLA alleles were included in this study, representing 204 common or well-documented (CWD) P groups (65.4% of all CWD P groups, version 2.0.0) (Table 1, S1 and S2 Tables) [13]. Homozygous genotypes comprised 15–19% of the genotypes at HLA-A/B/C/DRB1/DQB1 loci. Six null alleles from the current CWD catalog were included in the study, including A*24:11N, C*04:09N, DRB4*01:03:01:02N, DRB4*02:01N, DRB5*01:08N, and DRB5*01:10N. Four non-CWD null alleles, B*39:25N, B*15:26N, A*31:14N, and C*07:55N, were also represented in the study.

**Table 1:** Number of unique alleles, CWD P groups, non-CWD alleles in this study.

Locus	Count of unique alleles	CWD P groups	Total CWD P groups	Percent of all CWD P groups	Alleles outside of CWD P groups
A	62	33	57	57.9%	24
B	91	59	102	57.8%	30
C	40	24	38	63.2%	9
DPA1	6	3	3	100.0%	2
DPB1	31	12	14	85.7%	12
DQA1	16	7	7	100.0%	1
DQB1	18	14	17	82.4%	1
DRB1	60	40	62	64.5%	14
DRB345	17	12	12	100%	4
Total	341	204	312	65.4%	97

### Study design

**Fig 1:**
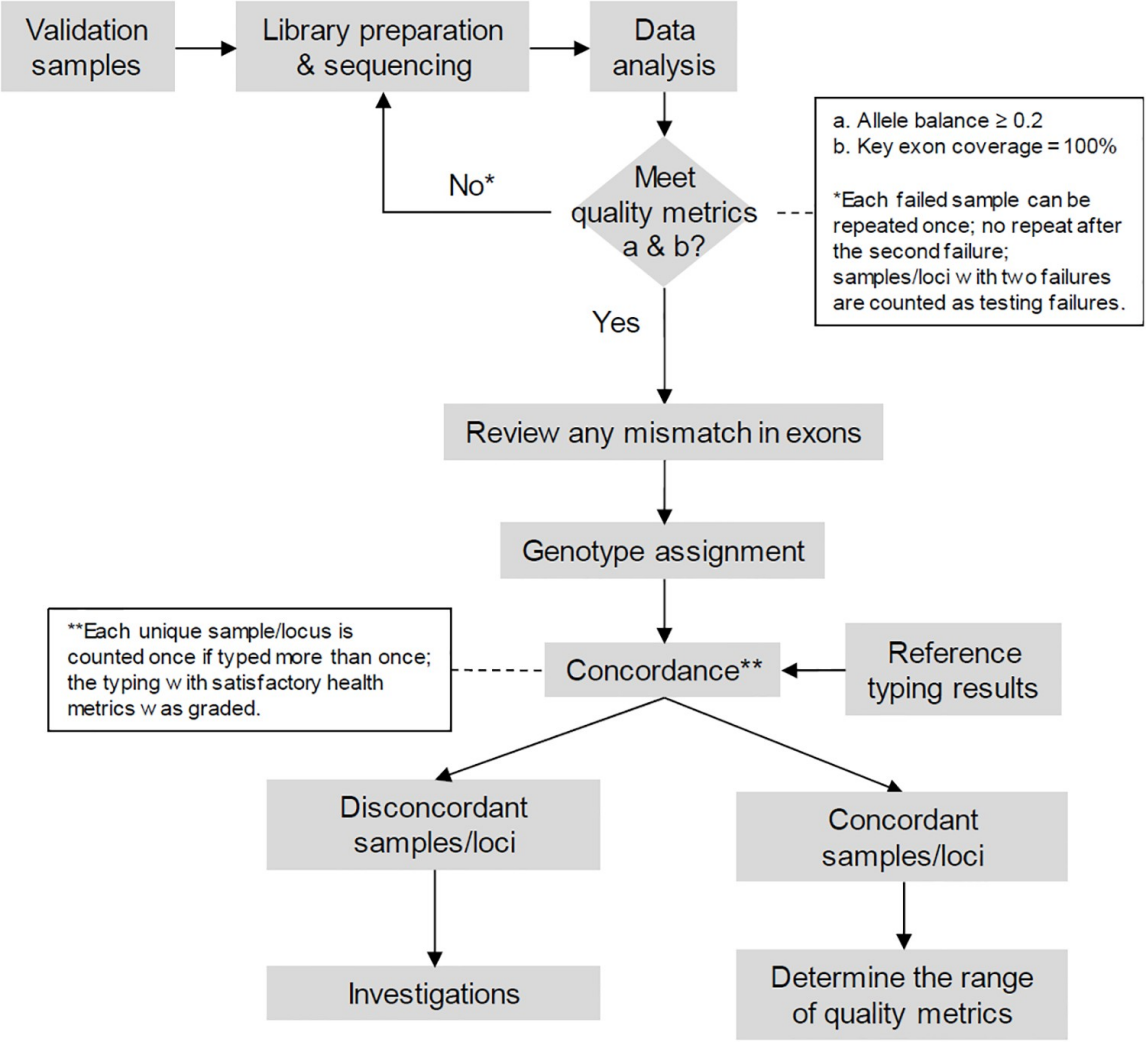
Study design.

We followed a pre-specified protocol shown in Fig 1 to ensure objective grading of the results. Samples must meet two health metrics to be graded for concordance: the allele balance must be 0.2 or higher, and the key exon coverage must be 100%. For failed samples, reanalysis was performed with an increased number of reads, if available, or the samples were typed for a second time. The primary endpoint of the study was the concordance between the AllType results and the reference results at the 2-field resolution. Technologists were blinded to the reference results during testing and data analysis. When ambiguity was encountered, we considered the National Marrow Donor Program (NMDP) policy to prioritize genotypes consisting of common or well-documented alleles (CWD). Discordant results were investigated to identify the potential cause. In addition, we determined the lower limit of genomic DNA concentration for the assay, the range of read coverage per sample and per locus, the range of key health metrics, and the ambiguity rate. Finally, we assessed the intra-run and inter-run reproducibility by testing three samples in triplicate and in four independent runs; these runs were performed by two technologists using three different lot of reagents. Different barcodes were used to index these reproducibility samples. We evaluated the concordance of the typing results and the correlation of key health metrics. Discordant samples were typed by TruSight HLA v2 and AlloSeq Assign (CareDx, Brisbane, CA) on the Illumina platform. The MinION device from Oxford Nanopore Technologies (ONT) was used to generate long reads to resolve cis-trans ambiguities in two discordant DPB1 genotypes.

### DNA extraction & quantification

Genomic DNA was extracted from blood specimens using the EZ1 DNA Blood 350 μL Kit (Qiagen, Hilden, Germany) on the EZ1 Advanced XL instrument. The proficiency testing cell lines (EBV-transformed B cells) from the University of California Los Angeles (UCLA) Immunogenetics Center, local buccal samples, and local biopsy specimens were extracted manually using the QIAamp DNA Mini Kit (Qiagen, Hilden, Germany).

### AllType workflow & HLA typing assignment

The AllType assay (One Lambda, West Hills, CA) amplifies and sequences five whole genes (HLA- A/B/C/DQA1/DPA1) and six near-whole genes (HLA-DRB1/DRB345/DQB1/DPB1; exon 1 and part of intron 1 not included) for HLA typing. Target amplification, library preparation, template preparation and chip loading on the Ion Chef, and sequencing on the Ion S5 instrument were performed following procedures specified by the vendor. Torrent Suite (Version 5.10.1) was used for basecalling, demultiplexing, and managing run-level and barcode-level data. TypeStream Visual (TSV, Version 1.2; Catalog ALL-11LX_001, ALL-11LX_007 and ALL-11LX_013; IMGT Version 3.33.0.0) was used to analyze locus-level data to determine HLA genotypes at 2-field resolution if associated health metrics were met. The default analysis parameters and health metrics thresholds recommended by the vendor were used throughout the study except that the allele balance at any locus must be 0.2 or greater, instead of the default cutoff value of 0.3.

### Data analysis

Concordance rates were reported at the sample, genotype, and allele levels. Homozygous genotypes were treated as two identical alleles at the same locus. DRB345 was treated as one genotype with up to two detectable alleles at one or two of these three loci depending on the haplotypes present in any one sample. Run-level quality metrics, including ion sphere particle (ISP) density, total reads, mean, median or mode read length, test fragment 50AQ17 and 100AQ17, were summarized for all ten runs in this study to define the ranges of these parameters. At the locus level, we examined the distributions of health metrics (key exon coverage, allele balance, uniformity, and mismatches in exons) and their impact on typing results.

## Results

### Concentrations of genomic DNA (gDNA), PCR amplicons, and individual libraries

**Fig 2:**
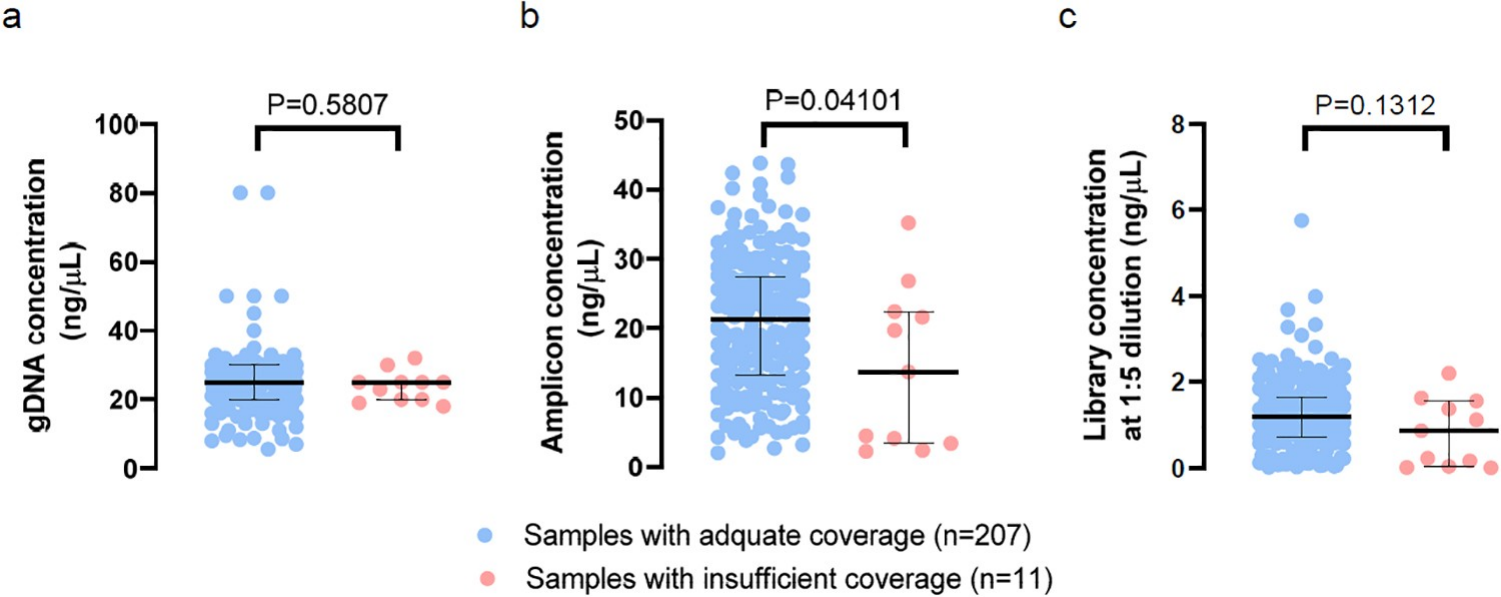
DNA concentrations throughout the AllType workflow. a) Genomic DNA (gDNA). b) PCR amplicons. c) Individual libraries (at 1:5 dilution). Concentrations for samples with adequate coverage for all loci (n = 207) or insufficient coverage for at least one loci (n = 11) are plotted separately. Black lines are medians and interquartile ranges.

The gDNA concentrations ranged from 5.6 to 80 ng/μL among all samples, and they were not significantly different between samples with adequate key-exon coverage (n = 207, median concentration: 25 ng/μL, range: 5.6–80 ng/μL) and those with inadequate coverage for one or more loci (n = 11, median: 25 ng/μL, range: 18–32 ng/μL) (Mann Whitney test [14], P = 0.58; Fig 2a).

The amplicon concentrations ranged from 2.0 to 43.8 ng/μL among all samples, and they were higher in samples with adequate key-exon coverage (median: 21.2 ng/μL, range: 2–43.8 ng/μL) compared with those with inadequate coverage (median: 13.7 ng/μL, range: 2.2–35.2 ng/μL) (P = 0.04; Fig 2b). However, lower amplicon concentrations could not reliably predict inadequate key-exon coverage. A receiver operating characteristic (ROC) analysis [15] showed an area under the curve of 0.682 (P = 0.042). Using < 5 ng/μL as the cutoff value, the sensitivity and specificity were 45.5% and 96.6%, respectively, for predicting inadequate key-exon coverage.

The library concentrations (at 1:5 dilution) ranged from 0.0076 to 5.76 ng/μL among all samples. Although lower library concentrations were enriched in the group with inadequate coverage, the library concentrations were not significantly different between samples with adequate key-exon coverage and those with inadequate coverage (P = 0.13; Fig 2c).

**Fig 3:**
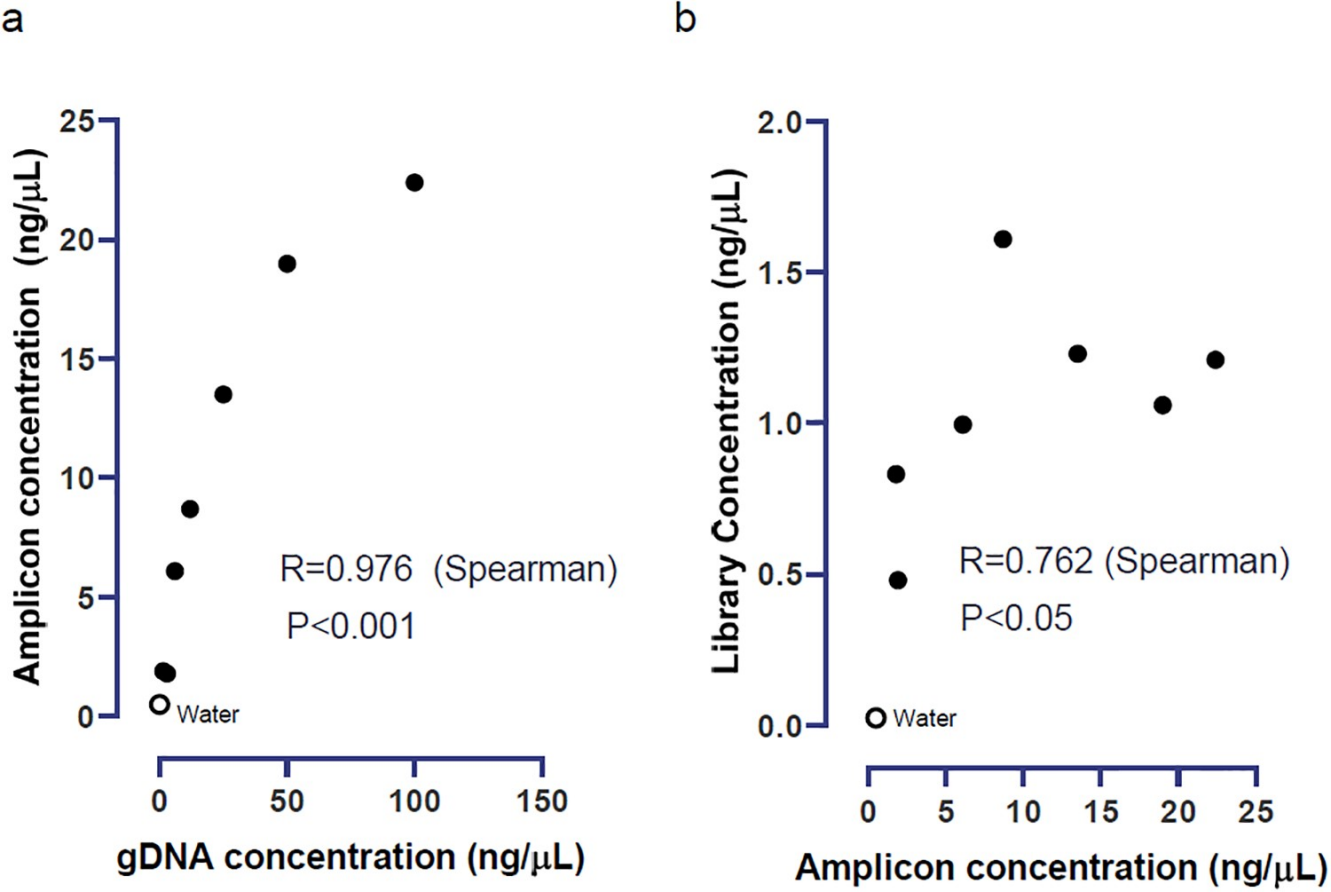
Concentrations of genomic DNA (gDNA), amplicons, and libraries in the titration study. a) Correlation between the gDNA concentrations and amplicon concentrations. b) Correlation between the amplicon concentrations and library concentrations.

We also performed a sensitivity analysis on the effect of input gDNA concentrations. Seven gDNA specimens from a serial 1:2 dilution of one sample were sequenced in one run. The concentrations ranged from 1.5 to 100 ng/μL, which was broader than the concentrations of the 218 regular samples. The PCR amplicon concentrations ranged from 1.8 to 22.4 ng/μL and correlated well with the input gDNA concentrations (Spearman R = 0.976, P<0.001; Fig 3a). The library concentrations (at 1:5 dilution) ranged from 0.48 to 1.61 ng/μL and correlated well with the amplicon concentrations (R = 0.762, P<0.05; Fig 3b). Concordant typing results were obtained at all input gDNA concentrations, and the negative control (water) was negative.

### Sequencing runs and run-level metrics

**Fig 4:**
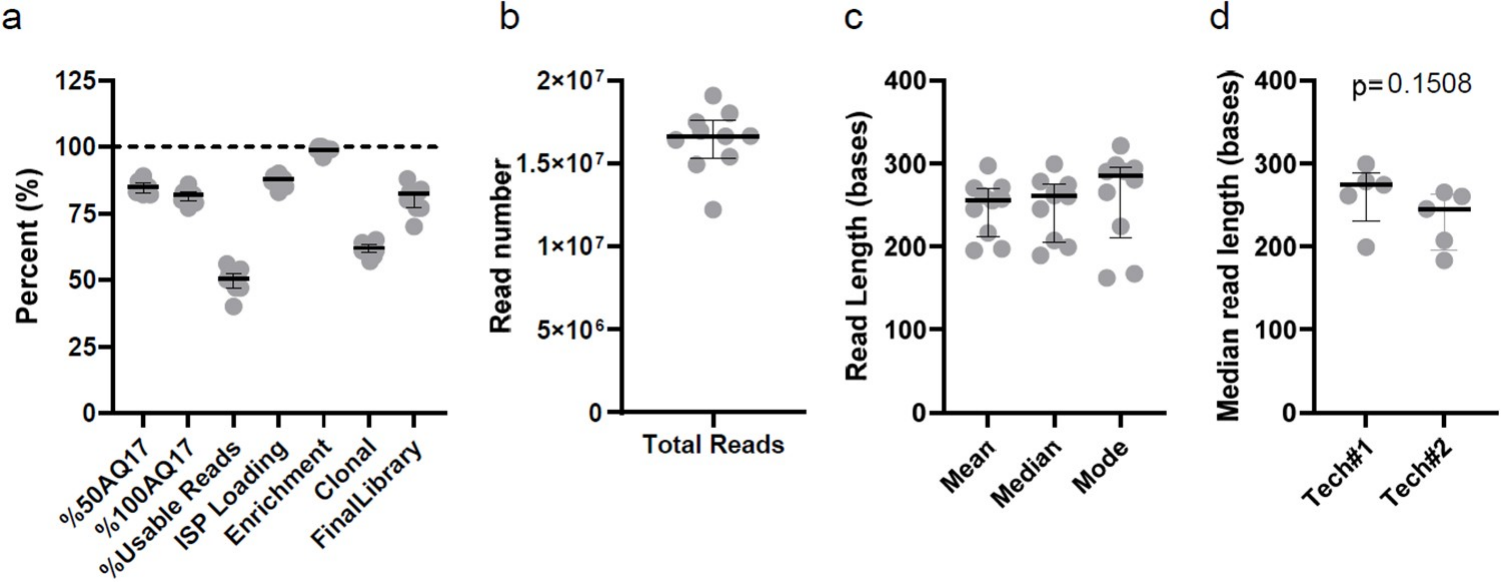
Distribution of run-level metrics from ten sequencing runs. a) Metrics for sequencing accuracy of test fragments (%50AQ17 and %100AQ17) and the percentage of reads usable for analysis. The latter is dependent on the percentages of ion-sphere particle (ISP) loading, template-positive ISP (enrichment), clonal ISP, and final library. b) Total reads usable for analysis. c) The mean, median, and mode of read lengths from 10 runs. d) The median read lengths stratified by technologists. Lines are medians and interquartile ranges.

Two technologists performed ten sequencing runs with 16–48 samples per run. The run-level quality metrics are summarized in Fig 4. We observed a narrow distribution of 50AQ17 (median: 85%, interquartile range or IQR: 82.7–86.3%) and 100AQ17 (82%, 79.8–83%), defined as the percentages of the test fragment (TF) sequencing reads with an error rate of 1 in 50 and a minimum length of 50 and 100 bases, respectively. The median percent usable reads was 50.5% (IQR: 47–52.5%), which was dependent on the percent chip loading (88%, 86.5–89%), percent enrichment (99%, 99–99.3%), percent clonal ion-sphere particles (62%, 60.5–63.3%), and final library (82.5%, 77–83.3%) (Fig 4a and 4b). The median read length observed in this study was 261 bases, and the coefficients of variations (CVs) for the mean, median, and mode of read lengths across all runs were 13.6%, 15%, and 21.6%, respectively (Fig 4c). The shorter median read lengths in three runs (approximately 200 bases) were not associated with any operator (Fig 4d). Importantly, there was no correlation between shorter read lengths and increased typing errors or ambiguity.

### Number of reads per sample and per locus

**Fig 5:**
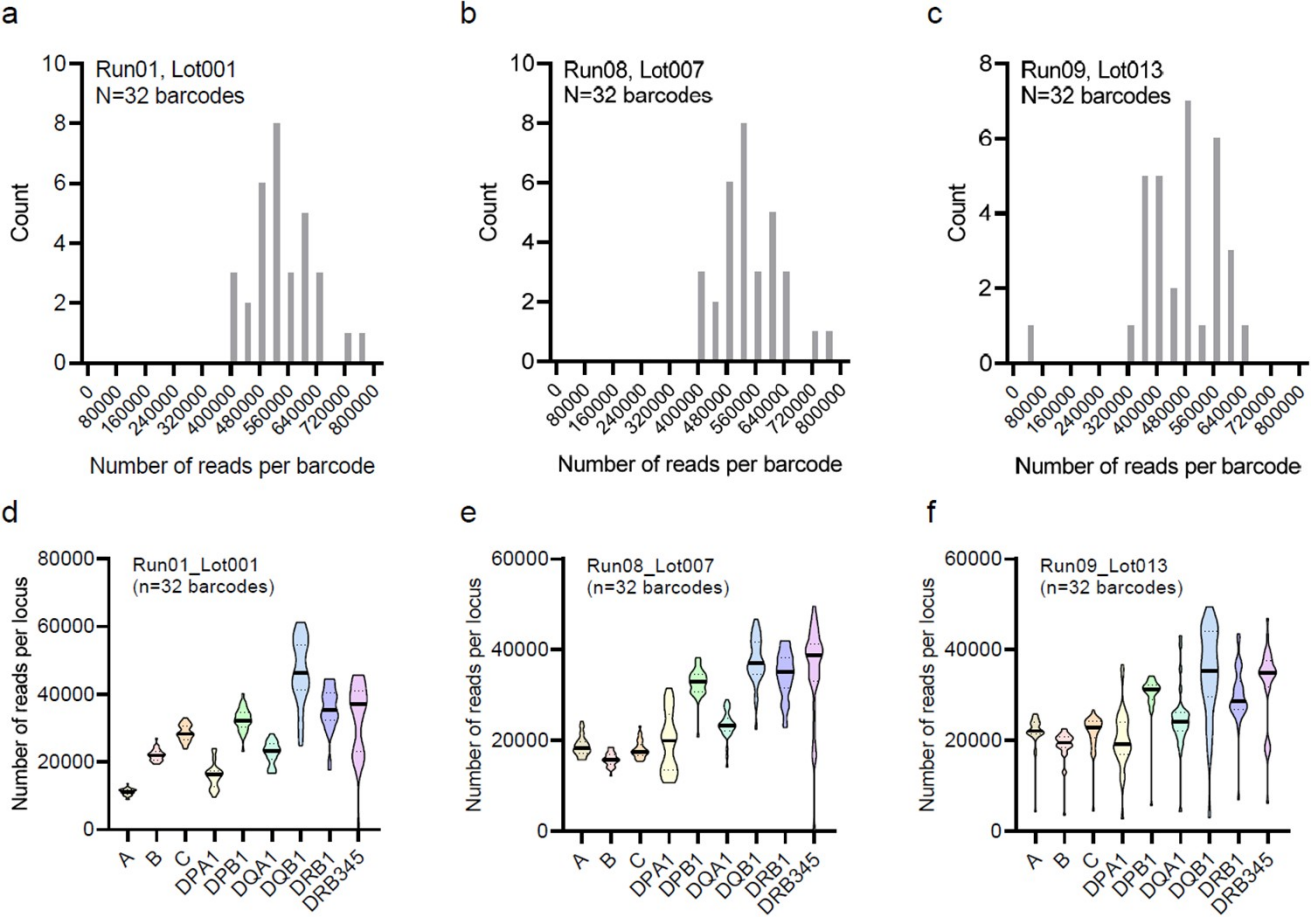
Distribution of the number of reads per sample and per locus. The histogram of read count per sample was plotted for three independent runs performed with three different lots of AllType reagents: a) Run01, Lot001. b) Run08, Lot007. c) Run09, Lot013. The number of reads per locus was also summarized for these runs: d) Run01, Lot001. e) Run08, Lot007. f) Run09, Lot013. Each run included 32 barcoded samples. Median (solid line) and interquartile (dotted line) are shown in the violin plots (d-f).

The number of reads per sample, after demultiplexing, showed a normal distribution in three representative runs (Fig 5a–5c), all including 32 samples per run but each using a different lot of AllType PCR primers. The mean reads number per sample ranged from 461,301 to 538,531 among these runs. The TSV software, by default, analyze up to 300,000 reads per sample for HLA typing. All but one sample in these three runs surpassed this threshold. The outlier in Run09 had 40,000 reads and was successfully typed with all health metrics met.

**Fig 6:**
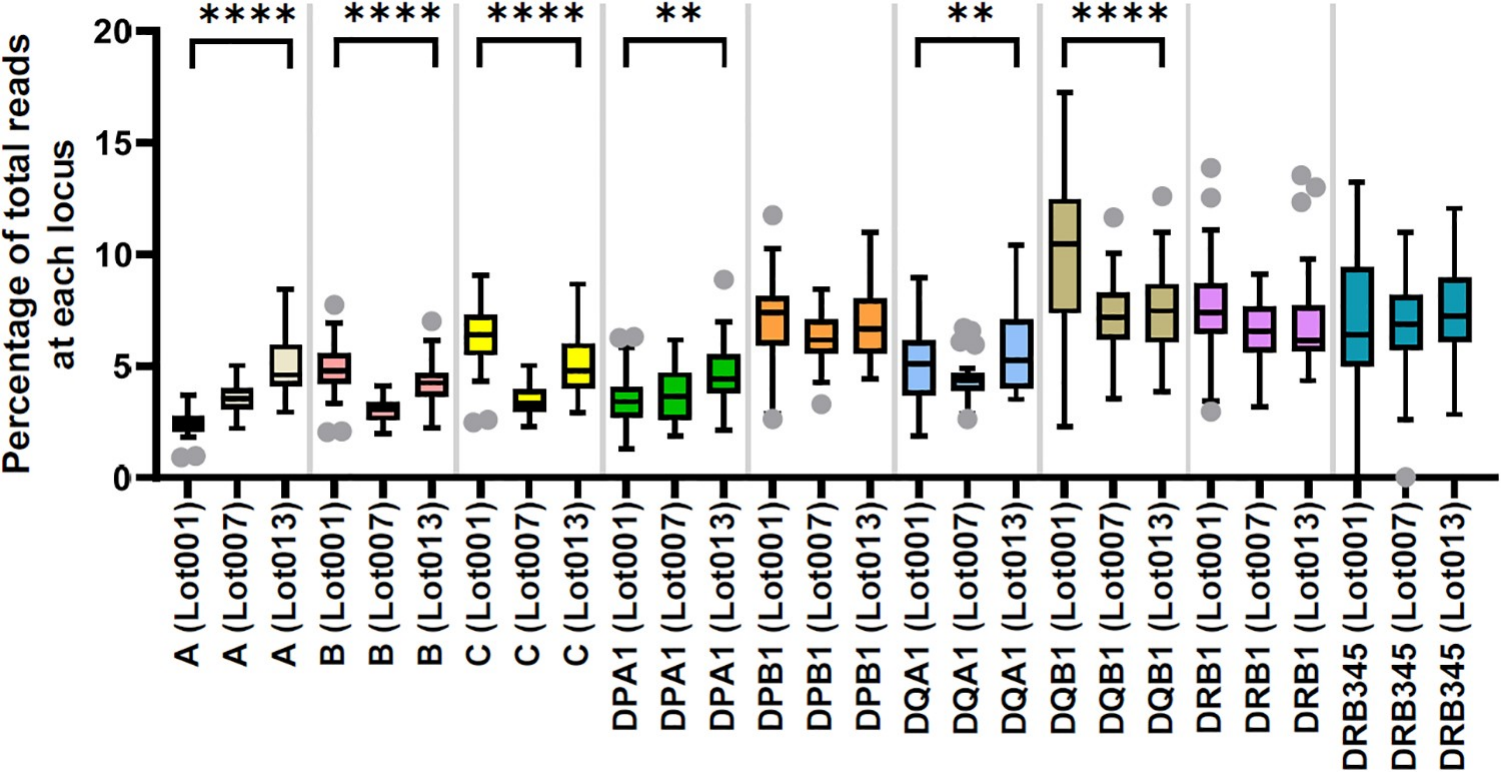
Inter-lot comparison of the percentages of total reads mapped to each locus. Each lot is represented by one run with 32 barcoded samples. Boxes represent the median and interquartile range (IQR); whiskers represent 1.5 IQR. ** P<0.01, **** P<0.0001, by ordinary one-way ANOVA.

We observed the following inter-locus and inter-lot difference in the number of reads mapped to each HLA locus (Fig 5d–5f). First, the number of reads per locus was higher for certain class II loci, i.e., DPB1, DQB1, DRB1, and DRB345, than class I loci in all three representative runs. This difference corresponds to the longer amplicons for these class II loci than those for class I loci. Second, the read number per locus was unbalanced among class I loci (A, B, and C) with the Lot 001 reagent (Fig 5d), while the inter-locus balance was improved with the Lot 007 and 013 reagents (Fig 5e and 5f). The imbalance among DPB1, DQB1, DRB1, and DRB345 was relatively modest. Third, we also normalized the number of reads per locus as a percentage of the total reads per sample and compared the values across different representative runs (Fig 6). Significant inter-lot differences were detected for A, B, C, DPA1, DQA1, and DQB1, which likely reflect reagent updates by the manufacturer to achieve more balanced coverage for these loci.

### Health metrics: Key-exon coverage, allele balance, and repeat testing

**Fig 7:**
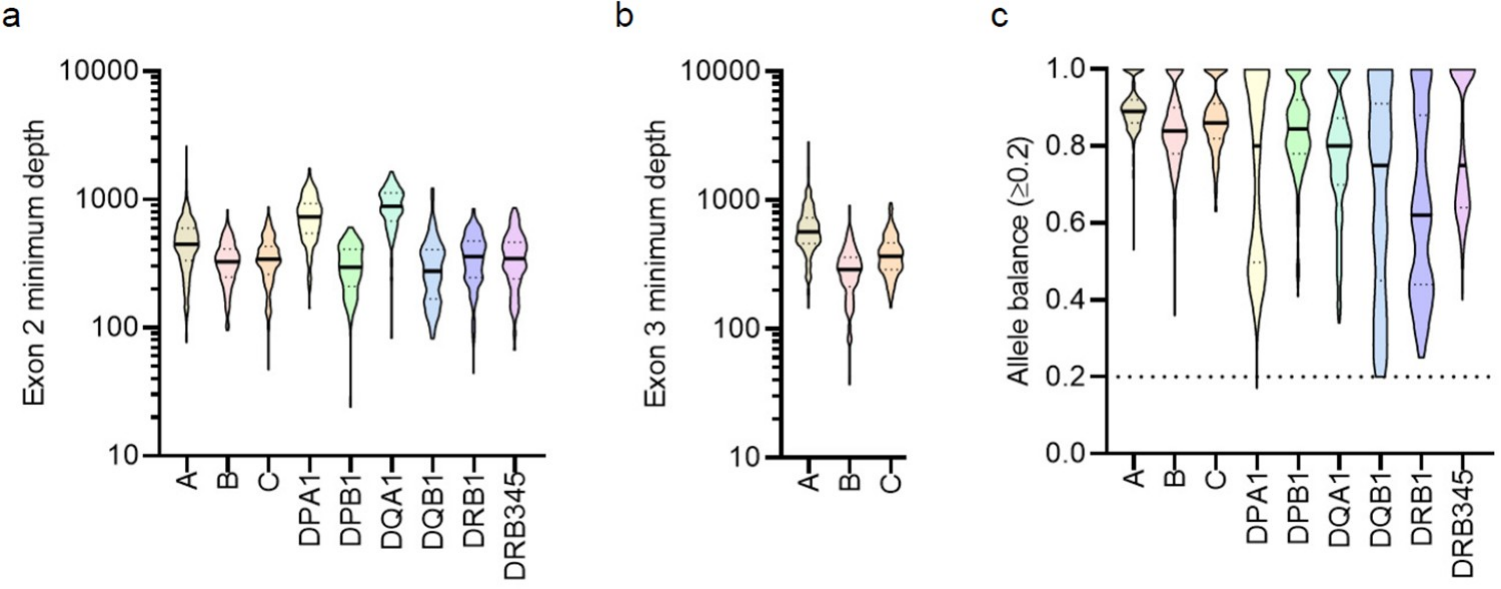
Distributions of minimum coverage depths in key exons and allele balance. a) Minimum coverage depth for exon 2 of all loci. b) Minimum coverage depth for exon 3 of class I loci. c) Allele balance. Values from 218 samples are plotted. For samples that underwent repeat testing, the values from the repeat testing (not the initial run) were included in the plots. Line and dotted lines in the violin plots represent median and interquartile range (IQR), respectively.

The minimum key-exon coverage was above 100 reads for most samples across all loci (Fig 7a and 7b), suggesting that excellent coverage can be achieved with 16–48 samples per run. For DPA1, DQB1, DRB1, and DRB345, the allele balance deviated from a normal distribution with a large number of samples affected by significant allele imbalance (Fig 7c).

Five samples had inadequate key-exon coverage, defined as less than 20 valid reads at any key-exon position for an allele, which was resolved upon reanalysis with additional reads (up to 600,000 per locus if available). Ten samples were re-typed due to inadequate coverage and unsuccessful reanalysis, giving a repeat testing rate of 4.6%. Seven of the ten samples passed all health metrics upon repeat testing. Three of the ten samples did not satisfy the key exon coverage for DQB1 in one sample and for DPB1 in all three samples. All three samples were PT samples stored at 4°C for over two years. Despite satisfactory gDNA concentrations (18–23 ng/μL), partial degradation of the specimens was observed on electrophoresis. Among all target loci, DRB1 (7 samples) was the most commonly affected by inadequate key-exon coverage. Only one sample had allele imbalance for DPB1 at the 4-field resolution, which did not affect reporting at the 2-field resolution, and the sample was not re-typed.

### Health metrics: Mismatches in exons

A total of 46 mismatches in exons were observed in this study, where the consensus bases from the sequencing reads differed from the reference alleles called by TSV (S3 Table). These mismatches were in non-key exons only, and most were found at the DRB5 locus (67.4%), followed by DPA1 (17.4%), C (8.7%), DQA1 (4.4%), and DQB1 (2.2%). The closest 2-field HLA alleles were assigned as the final typing for comparison with the reference typing results.

Thirty two of the 46 exon mismatches (69.6%) were likely sequencing artifact (S3 Table). The G>T variant at exon 6 position 12703 (gDNA alignment position, E6-12703) of DRB5*01:01:01 was the most common mismatch in exons (n = 21) observed in our study, followed by the same mismatch with DRB5*01:02 (n = 4) and DRB5*02:02:01 (n = 4). These mismatches occurred primarily with Lot 001 and 007 reagents, but not Lot 013, and their interpretation was confounded by high background at the position suggestive of aberrant read mapping. Three mismatches occurred in homopolymer regions in three samples, and the supporting reads for these mismatches were all in one direction rather than bidirectional.

Fourteen of the 46 exon mismatches were supported by the sequencing reads (S3 Table). Except for two synonymous mutations, 12 mismatches were missense mutations that were predicted to cause substitutions in the protein sequence outside of the antigen recognition domains.

### Concordance with reference typing and investigation of discordant results

The AllType typing results were concordant with all evaluable genotypes in 215 of the 218 samples, with a sample-level concordance rate of 98.6%. The overall concordance rates at the genotype and allele levels were 99.8% and 99.9%, respectively. Detailed results for each HLA locus are reported in Table 2. The three discordant genotypes and our investigation are described in Table 3. The discordant DRB1 genotype involved alleles in the same G group and could be explained by a single-base variant in exon 4 that was missed by TSV. The G group genotype, homozygous DRB1*09:01:02G, was in agreement between AllType and the reference. The two discordant DPB1 genotypes also share the same G group typing, DPB1*04:01:01G and DPB1*04:02:01G, and the discrepancies were due to cis-trans ambiguity. Both samples were PT samples from 2016 and 2017, and the reference typing results appeared to have considered the key exon (exon 2) only.

**Table 2:** Concordance at the genotype and allele levels.

Locus	Genotype-level evaluation			Allele-level evaluation		
	Evaluable genotypes*	Concordance	Concordance rate	Evaluable alleles*	Concordance	Concordance rate
A	218	218	100.0%	436	436	100.0%
B	218	218	100.0%	436	436	100.0%
C	218	218	100.0%	436	436	100.0%
DPA1	133	133	100.0%	266	266	100.0%
DPB1	151	149	98.7%	302	298	98.7%
DQA1	163	163	100.0%	326	326	100.0%
DQB1	217	217	100.0%	434	434	100.0%
DRB1	217	216	99.5%	434	433	99.8%
DRB345	153	153	100.0%	306	306	100.0%
All loci	1688	1685	99.8%	3376	3371	99.9%

* Loci with unsatisfactory health metrics were excluded, including DQB1 in one sample, DPB1 in three samples, and DRB1 in one sample.

**Table 3:** Discordant results and further investigation.

Run-ID	Barcode	Locus	Reference typing	AllType result	Investigation
3	55, 62	DPB1	DPB1*04:01 DPB1*04:02	DPB1*105:01 DPB1*126:01 OR DPB1*665:01 DPB1*126:01	All health metrics were met. The two genotypes by AllType were due to a sequence variant in exon 1 that was not covered by the assay. The reference and AllType genotypes indicated a cis-trans ambiguity across exons 2 and 3. The ambiguity was not resolved by alternative NGS methods on the Illumina platform. Long reads spanning exons 2 and 3 generated by nanopore sequencing were consistent with the reference typing.
6	40	DRB1	DRB1*09:01 DRB1*09:21	DRB1*09:21 DRB1*09:21 OR DRB1*09:21 DRB1*09:31 OR DRB1*09:31 DRB1*09:31	All health metrics were met. DRB1*09:01:02:01 has one base difference from DRB1*09:21 in exon 4 (E4-8874). DRB1*09:31 has a reference sequence that covers exons 1–3 only; it has one base difference from DRB1*09:01:02:01 and DRB1*09:21 in exon 1. DRB1*09:01 was not included in the genotype by AllType because the supportive reads were considered as high background rather than true signal. This seemed to be an idiosyncratic event unique to this sample, which was typed correctly by a newer version of TSV.

We typed the discordant samples by TruSight HLA [10] and AlloSeq Assign (CareDx, Brisbane, CA) assays on the Illumina platform. The DRB1 typing by both Illumina-based methods agreed with the reference. Neither Illumina-based method resolved the cis-trans ambiguity of the two DPB1 genotypes. Nanopore sequencing generated long reads capable of phasing the distant variants, which supported the genotype of DPB1*04:01 and DPB1*04:02.

### Ambiguity

Ambiguity was encountered in 112 samples for 144 genotypes, and three categories were described as follows. First, 116 ambiguous genotypes (80.6%) involved DPB1, DQB1, DRB1, and DRB345 genes and were explained by the lack of exon 1 and intron 1 coverage. Second, 26 ambiguous genotypes (18.1%) were due to cis-trans ambiguity across different exons. All alternative genotypes included one or two rare alleles, which did not need to be resolved per current NMDP policy and could be reported using existing NMDP codes. Third, ambiguous genotypes due to intronic variant were frequently identical at the 2-field resolution except in two cases (1.4%), where B*27:05:02:04Q was listed as an alternative allele. B*27:05:02:04Q has an A at the 3’ end of intron 2, substituting a commonly observed G; the variant is predicted to disrupt splicing and result in an altered protein sequence. Upon inspection of the reads mapped to the region, B*27:05:02:04Q was ruled out in both cases due to the lack of read support.

### Intra-run and inter-run reproducibility

Three samples for reproducibility evaluation were tested in triplicate in four runs, a total of 12 repeats for each sample. All genotypes were concordant among all repeats with the following two exceptions. One repeat of sample #1 did not meet the minimum reads requirement and was excluded from analysis. Sample #3 had allele imbalance for DRB1 in four out of 12 repeats, which was also excluded from analysis.

To assess the reproducibility of key health metrics, we first examined the pairwise correlation of mean exon 2 coverage across all loci between repeats of each sample. The correlation of 34 pairs of intra-run repeats showed Pearson coefficients ranging from 0.7 to 1. The inter-run correlations were lower than intra-run correlations with 17 out of 153 Pearson coefficients below 0.4, an arbitrary threshold for acceptable correlation. Exclusion of DQB1 from this analysis improved all coefficients to 0.4 or higher. We also examined the pairwise correlation of allele balance across all loci between repeats of each sample. The correlation of intra-run repeats showed coefficients ranging from 0.4 to 1. The inter-run correlations were, again, lower than intra-run correlations with 32 out of 153 Pearson coefficients below 0.4. Exclusion of DRB1 from this analysis improved all coefficients to 0.4 or higher.

## Conclusion & discussion

This study established an excellent accuracy and ambiguity profile for the AllType workflow in 2-field HLA typing. We included genetically diverse samples of multiple specimen types that are routinely tested in a hospital-based HLA laboratory. We also implemented a pre-specified policy for repeat testing and concordance grading to ensure an objective evaluation. The assay tolerated a broad range of input DNA concentrations and individual library concentrations with a low repeat testing rate. The number of reads per sample and locus were adequate for the application. With the multiplexed PCR design, we observed some inter-lot variations in the per-locus reads number, as well as a trend towards a more balanced inter-locus coverage with later lots of reagents. We also observed increased exon mismatches with earlier lots but not a later lot of reagent (S3 Table), which underscored the need to characterize these mismatches during data analysis and to monitor their occurrence for quality assurance purposes. With satisfactory key exon coverage and allele balance, 99.8% of the AllType genotypes were concordant with the reference results. For the three discordant genotypes, the AllType results were clinically acceptable in all three cases, where the G group typing results agreed with the reference.

Our findings confirm the recent report by Cargou and colleagues on the performance of the AllType workflow [11]. We also present additional information on the accuracy of 2-field typing for DQA1 and DPA1, which may be beneficial for solid-organ donor typing [16]. Moreover, we describe the distributions of key health metrics, gDNA and library concentrations, and reads number per sample and per locus. These data are valuable for quality monitoring, decision-making on repeat typing, and troubleshooting in times of testing failure. The minimum guidelines on the validation of NGS HLA typing by the American Society for Histocompatibility and Immunogenetics (ASHI) also emphasizes the evaluation of health metrics associated with NGS workflows in clinical HLA laboratories. Related to the report by Cargou and our study, Barone and colleagues reported the satisfactory performance of a similar workflow on the Ion-torrent platform, NXType [12], which features two separate PCR reactions for class I and II HLA genes, respectively. De Santis’s group also reported a highly accurate methods based on the Ion-torrent sequencer, and four PCR reactions were used to amplify a total of 11 HLA genes [17].

The “long-amplicon, short-reads” approach to NGS-based HLA typing has also been implemented successfully on the Illumina platform. Several commercially available assays have achieved comparable accuracy and efficiency based on well-designed validation studies [9, 10, 18–20]. The general workflows of these assays are similar to AllType, except for some differences in the PCR strategy, barcoding method, and sequencing chemistry. Typical for these assays, target HLA genes are amplified separately and then indexed with barcodes for each locus or each sample. For example, the Omixon assay analyzes up to 40,000 reads for locus-indexed libraries and 200,000 reads for sample-indexed libraries [9]. With the Illumina sequencing chemistry, the sequencing reads have a lower error rate than reads from the Ion-torrent sequencer; the paired-end reads with longer library fragments may theoretically enable the phasing of more distant variants. Nevertheless, Ion-torrent S5 has a shorter sequencing time of approximately 7 hours; the higher error rate and its typical read length of 200–300 bases appear to be inconsequential for the AllType application.

Our study has a few limitations. First, this is a single-center, single-platform evaluation with a modest sample size. While we provided in-depth information on the AllType assay, other multi-center, multi-platform comparison studies are invaluable in contrasting the performance of different NGS assays relative to each other [21, 22]. Second, our study did not evaluate HLA typing beyond the 2-field resolution. Two-field HLA typing remains the gold standard for recipient-donor matching in allogeneic hematopoietic stem cell transplantation [23]. Few clinical laboratories are currently reporting 3-field or 4-field HLA typing, although a potential role for this ultra-high resolution (UHR) typing is emerging in several recent studies [24, 25]. One potential barrier for the validation of UHR typing is the availability of clinical samples with known 3- and 4-field typing; some reference cell lines typed recently at UHR [21, 26] may be an acceptable alternative. Third, we handled exon mismatches by in-depth review of read mapping data and did not attempt to verify them by a different method. No mismatch occurred in key exons during our study; the closest alleles in the HLA/IMGT database [27] were assigned and used for concordance grading. We took this pragmatic approach to mimic the intended routine practice in our laboratory. Our current standard operating procedure also requires that mismatches in key exons that change the protein sequence must be verified by an orthogonal method.

The future landscape of NGS-based HLA typing will be defined by continued innovation and clinical investigation. Additional technologies such as the single molecule real-time (SMRT) sequencing on the Pacific Biosciences’ platform has been effectively employed for HLA typing [26, 28, 29]. The technology enables the “long-amplicon, long-reads” approach, which allows the long-range amplicons to be sequenced as a whole without fragmentation. This feature simplifies the library preparation and enhances the capability of phasing distant variants. Oxford Nanopore Technologies may offer another option for implementing the “long-amplicon, long-reads” approach for HLA typing [30–32], although better bioinformatics tools are needed to overcome the high error rate of the nanopore sequencing reads. On the other end of the spectrum, Lange and colleagues took the “short-amplicon, short-reads” approach and developed a workflow to sequence selected exons only on the Illumina platform. With the bypass of conventional library preparation and a high degree of automation, this method maximized the efficiency for donor registry HLA typing [33, 34]. Together with AllType and other Illumina-based assays discussed above, HLA laboratories will have many good options to meet their diverse clinical needs.

## Supporting information

S1 Table Genotypes of 218 unique samples. (XLSX)

S2 Table Alleles not in the CWD P group list. (XLSX)

S3 Table Mismatches in exons. (XLSX)
